# Ibuprofen versus paracetamol for treating fever in preschool children in Nigeria: a randomized clinical trial of effectiveness and safety

**DOI:** 10.11604/pamj.2020.36.350.21393

**Published:** 2020-08-26

**Authors:** Ekaete Olajide Alaje, Ekong Emmanuel Udoh, Patrick Aboh Akande, Friday Akwagiobe Odey, Martin Madu Meremikwu

**Affiliations:** 1 Paediatric Department, Lagos State University Teaching Hospital, Ikeja, Lagos State, Nigeria,; 2 Paediatric Department, College of Health Sciences, University of Uyo, Akwa Ibom State, Nigeria,; 3 APIN Public Health Initiatives, Abuja, Nigeria,; 4 Paediatric Department, College of Medical Sciences, University of Calabar, Calabar, Nigeria

**Keywords:** Ibuprofen, paracetamol, fever, underfives, trial, effectiveness, safety

## Abstract

**Introduction:**

fever is the primary symptom of most childhood illnesses and a cause of concern to their caregivers. The antipyretics commonly used to treat fever are ibuprofen and paracetamol. Most studies on the effectiveness of ibuprofen and paracetamol in treating fever in under-fives were conducted in Europe and North America with very few in African children. This study was aimed at assessing the effectiveness and safety of a single dose therapy of ibuprofen versus paracetamol for treating childhood fever in Nigeria.

**Methods:**

a randomized, controlled clinical trial was conducted in the University of Calabar Teaching Hospital, in Nigeria. A total of 140 eligible children aged 6-59 months with tympanic temperature of 38°C-40°C were enrolled, and 70 of them were assigned to one arm that received a single dose of ibuprofen (10mg/kg) and 70 had paracetamol (15mg/kg). After drug administration, the children were admitted and observed in the hospital for six hours during which period a half-hourly temperature measurement and monitoring for adverse events were done.

**Results:**

the overall result showed that ibuprofen had a better fever reducing effect compared to paracetamol. The proportion of afebrile children in the ibuprofen versus paracetamol group at 1.5-2.5 hours of administration of the drugs was statistically significant (p = 0.04). The adverse events of both drugs were mild and quite comparable with vomiting being the commonest.

**Conclusion:**

ibuprofen is more effective in the treating fever in under-fives compared to paracetamol. The adverse events of both drugs were mild and comparable.

## Introduction

Fever is a common symptom of illness in preschool children. It is a major reason for clinic visits and cause of concern to caregivers. According to the 2010 Nigeria Malaria Indicator Survey, the prevalence of fever in children under five years of age in Nigeria is estimated to be 35% [1]. It is responsible for about 25% of visits to paediatric emergency rooms [2]. In sub-Saharan Africa, infectious diseases like malaria, acute respiratory infections including pneumonia, diarrhoea, measles and tuberculosis are the most commonly reported causes of fever in preschool children [3]. These diseases are estimated to cause about 68% of deaths among preschool children [4]. Fever could disrupt sleep, activity, appetite, comfort, lead to school absenteeism and febrile convulsion in preschool children. For these reasons, caregivers of preschool children institute various measures to treat fever. Opinions are divided on the temperature at which fever should be treated [5]. The Integrated Management of Childhood Illness guidelines recommend that children with axillary temperatures greater than 38.5°C be given antipyretic to relieve discomfort and common symptoms associated with fever [6]. However, caregivers of preschool children often seek treatment for fever even at temperatures below 38.5°C to avert the likely occurrence of adverse outcomes associated with fever [7].

The commonly prescribed pharmacological agents for treating fever are paracetamol and ibuprofen. paracetamol has been used over the years for treating fever in children while the use of ibuprofen is relatively recent [8,9]. Both drugs are commonly used in Europe and North America to treat fever in preschool children because they have been shown to be efficacious and safe [10]. The common adverse events reported with paracetamol in treating childhood fever are nausea, diarrhea and pruritic rashes while those of ibuprofen are nausea, vomiting and abdominal pain [11,12]. In Nigeria and most parts of sub-Saharan Africa, the treatment of fever in this age group is mainly with paracetamol [7]. There is paucity of comparative data on their use in African children. This study was aimed at comparing the effectiveness and safety of ibuprofen versus paracetamol in the treatment of fever among preschool children in Nigeria.

## Methods

The study was an open label randomized clinical trial that compared the effectiveness and safety of a single-dose of ibuprofen (10mg/kg) versus paracetamol (15mg/kg) over a period of 72 hours in febrile pre-school children. The study was conducted in the Paediatrics Department of the University of Calabar Teaching Hospital (UCTH), Calabar, Cross River State of Nigeria between December 2014 and May 2015. Calabar is the capital of Cross River State in Nigeria with an estimated population of 375,196 [13]. The residents of Calabar are largely farmers, fishermen, wood loggers, traders, civil servants, and students.

**Sample size determination:** the sample size was estimated based on the primary outcome measure, i.e. the mean reduction in temperature. The following formula was used to estimate the required size at a significance level of 5% and power of 80% [14].

$$N=\frac{(r+1){\left(Z_{\sigma /2}+Z_{1-\beta }\right)}^{2}\sigma^{2}}{rd^{2}}$$

Where: N = total sample size for the study i.e. n_1_+ n_2_; Zσ = normal deviate at a level of significance (1.96 at 5% level of significance); Z_1-β_= normal deviate at 1-α% power (0.84 at 80% power); r = n1/n2 (the ratio of sample size required for the two groups; equals 1 in the case of this study due to equal sample sizes); σ = pooled standard deviation (SD) of mean reduction in temperature in the two groups; d = difference of means of the reduction in temperature in the two groups. For this study, pooled SD of the mean temperature reduction of 0.9°C was adopted from a previous study by Vyas *et al*. [15]. The clinically relevant difference in temperature reduction measured over six hours after administering the first doses of ibuprofen and paracetamol was taken as 0.3°C [10]. Based on the above, the minimum sample size was determined to be 127. This was increased to 140 to account for an attrition rate of 10%, with 70 participants in each arm of the study.

**Ethical issues:** ethical clearance for the conduct of this study was obtained from the Health Research Ethics Committee of UCTH. There was strict adherence to Good Clinical Practice (GCP) including observation of articles of Helsinki concerning biomedical research and human rights [16,17]. Adequate information about the study was given to the caregivers of the eligible who were enrolled into the study after parental consent had been obtained.

**Study population:** this consisted of febrile children aged six to 60 months seen in the Children Emergency Room (CHER) or the Children Outpatient Clinic (CHOP) of the UCTH.

**Eligibility criteria:** these were children aged six to 60 months with a tympanic temperature 38-40°C on presentation without any of the following; convulsion, repeated vomiting, hyperpyrexia, otitis media, chronic illness or ingestion of antipyretics six hours prior to presentation.

**Clinical assessment:** the biodata of the enrolled children were obtained. This was followed by the history of present illness, medications taken for the fever, history of antipyretic ingestion six hours prior to enrolment in the study and the past medical history. General physical examination, anthropometric measurements and systemic examination were performed.

**Temperature measurement:** tympanic temperature was measured with a Medline® Ear Thermometer (Medline Industries, Inc., Mundelein, IL 60060 USA, MDS9700) by gently pulling the ear back to straighten the ear canal and then positioning the probe into the ear canal aiming towards the membrane of the ear drum to obtain an accurate reading. It was kept there until it beeped and the temperature was then read and recorded immediately in the child’s case report form. Temperature was measured three times with the same ear. If the three measurements were different, the highest reading was taken. The tympanic thermometer was used in this study because it is convenient, takes a shorter time to obtain a reading and is more sensitive in measuring the core body temperature when compared to conventional thermometer [18].

**Randomization and allocation of participants:** a stratified randomization method based on age and sex was used to assign participants into the intervention groups. Age and sex were considered to be potential confounders and were therefore used to create strata for patient allocation. Patient attendance records were used to estimate the age ratios of febrile children aged 6 months to 60 months who presented with fever at the Children’s Emergency Room and Children’s Outpatient Clinic in the preceding one-year period. These ratios and the pre-determined sample size of 140 were used to stratify the patients. There was a total of 70 children in each group, with an equal number of males (35) and females (35). The male and female subgroups were further stratified, with each sex having the following number of children by age group: 6-12 months (10 children), 13-24 months (8), 25-36 months (7), 37-48 months (5), and 49-60 (5). The sequentially numbered, opaque, sealed envelopes (SNOSE) method was used to conceal the allocation of the children to the groups from the research assistants and patient’s caregivers [19]. File jackets were prepared for each stratum blue colour for the males and pink colour for females. The different files were then labelled with the stratum age category and sex. The file jackets were kept in two separate boxes according to their colours. Each file jacket had envelopes corresponding to the number of participants in the stratum. Each envelope contained an allocation paper which had identifier (A or B) printed on it for allocation to each of the two treatment groups- ‘A’ for paracetamol and ‘B’ for ibuprofen. This allocation paper was concealed by first wrapping it with foil paper before inserting it into the envelope. The set of envelopes for each file jacket were thoroughly shuffled and sequentially numbered to make for an unpredictable allocation sequence. The research assistant allocated children in each stratum (according to the age and sex) to treatment groups by picking an envelope from the file jacket for that stratum. The envelope was then opened to reveal the allocation paper. The patient identification number and allocation group were then entered on the data collection tool.

**Investigational products:** the investigational products were ibuprofen (Brustan-N® syrup) and paracetamol (Emzor paracetamol® syrup) The ibuprofen syrup was manufactured by Ranbaxy Nigeria Ltd with NAFDAC registration number A4-0488; batch number 2660885; manufacturing date: Dec/2014; expiring date: Nov/2016. Each bottle contained 60ml of the medication with 100mg/5ml. The paracetamol was manufactured by Emzor Pharmaceutical Industries Ltd. with NAFDAC registration number 04-0289; batch number L1004T; manufacturing date: Sept/2014; expiring date Sept/2017; each bottle contained 60ml of the medication with 125mg/5ml. The drugs were obtained from the UCTH Pharmacy and were stored in a cabinet at optimum temperature.

**Treatment and follow up:** treatment was administered by the study nurse based on the body weight and intervention arm of the participants. Those in the paracetamol group received 15mg/kg/dose of the drug while those in the ibuprofen group had 10mg/kg/dose of the medication. The baseline temperature of the children at the time of commencement of treatment was recorded after which the tympanic temperature was recorded half hourly till the end of six hours being the efficacy endpoint of the study [20,21]. Each child also received concomitant treatment for the diagnosed underlying cause of the fever. The children were observed for occurrence of adverse events by the study team over the six hour period. The caregivers were educated on adverse events and asked to report same if noted after discharge. First 72 hours were by active follow-up during which phone calls were made to ask about the adverse events and subsequently by passive follow up wherein caregivers reported any event observed.

**Data analysis:** statistical package for the social sciences (SPSS) version 21 was used for data analysis. The children’s characteristics were described using frequency and percentage for categorical variables and means for continuous variables. Categorical variables were compared using Pearson’s chi-square test (χ^2^) while Independent t-test was used for comparing continuous variables. The mean reduction in temperature at different time points was demonstrated as the area under the curve (AUC) on a chart, and this was compared between both groups. The comparison of the means of continuous outcome variables was done using Independent t-test and the effect size produced by the drugs determined. The proportions of categorical outcomes were compared between both groups using z test. The odds of developing adverse events was also determined. Statistical tests were significant if p-value was < 0.05.

## Results

**Figure 1:**
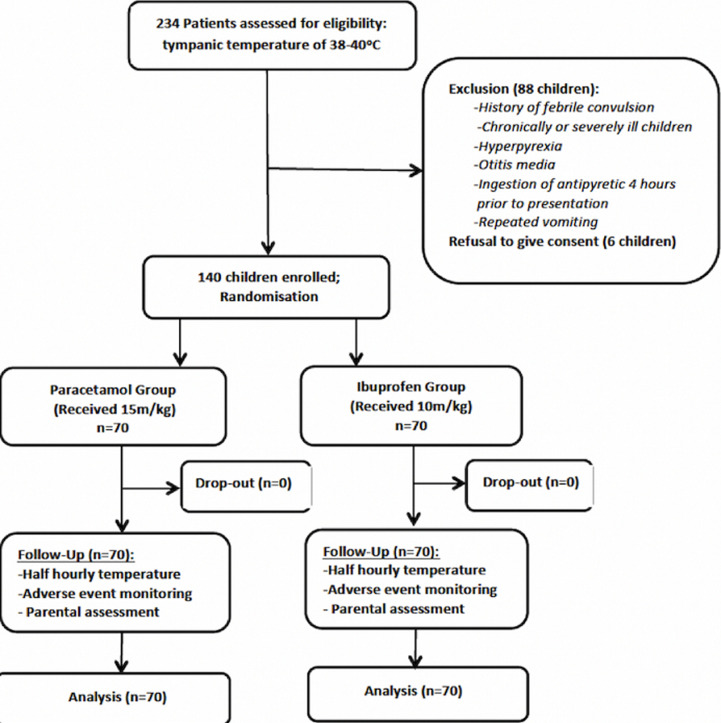
clinical trial flow diagram

A total of 234 children between the ages of six and 60 months with fever were assessed for enrolment in the study. Of this number, 140 eligible children whose parents gave informed consent were randomized into two treatment arms of 70 children each. There was no loss to follow-up during the six-hour period of observation post-administration of the drugs as shown in the CONSORT study flow diagram (Figure 1) [19].

**Baseline characteristics of study participants:** a total of 140 children aged six months to 60 months were recruited into the study. Of this number, 70 were males and 70 females with a male: female ratio of 1: 1. The children were randomized to receive either Syrup paracetamol (15mg/kg) or ibuprofen (10mg/kg) as a single dose therapy. Equal numbers of boys (35) and girls (35) were allocated to each of the intervention groups, which gave 70 children each in the paracetamol and ibuprofen groups (total=140). The baseline characteristics of the children were not statistically different between both groups, as displayed in Table 1.

**Table 1:** demographic and baseline characteristics of both group

Variable	Paracetamol Group (N=70)	Ibuprofen Group (N=70)	Test value	95% CI	p-value
Sex			χ2= 0	-0.16, 0.16	1.00
Male, n (%)	35 (50)	35 (50)			
Females, n (%)	35 (50)	35 (50)			
Mean age ± SD,mo	26.7 ± 16.8	26.6 ± 16.2	t=0.20	-5.47, 5.49	0.98
Mean body weight ± SD, kg	12.2 ± 4.52	12.4 ± 5.03	t=-0.34	-1.87, 1.33	0.74
Mean height ± SD, cm	26.7 ± 16.8	26.6 ± 16.2	t =-0.91	-8.78, 3.25	0.37
Mean baseline temperature, °C	39.13 ± 0.58	39.23±0.68	t=-0.86	-0.30, 0.12	0.39

**Mean temperature reduction produced by the drug at different time points:** the mean reduction in temperature was more in the ibuprofen group than the paracetamol group at all the time points as shown in Table 2. The difference in the mean temperature reduction between both groups was greatest at 3.5 hours (0.29°C) and least at 0.5 hours (0.02°C). Furthermore, the difference in the half-hourly mean temperature reduction between both groups was statistically significant from 2.0 hours to 4.5 hours after drug administration as shown in Table 2. In addition, at the end of the six hour period, a small effect size of 0.13 was produced. This was calculated using the formula, Effect Size, d = m1- m2/pooled sd. Where m1 and m2 were the mean temperature reduction in the ibuprofen and paracetamol groups respectively; sd1 and sd2 being the standard deviations of the mean temperature reduction in ibuprofen and paracetamol groups respectively.

**Table 2:** comparison of mean temperature reduction at different time points

	Mean temperature reduction ± SD, °C				
Time, hr	Paracetamol group (n=70)	Ibuprofen group (n=70)	t- value	95% CI	p-value
0.5	0.36 ± 0.35	0.38 ± 0.38	-0.35	-0.14, 0.10	0.75
1	0.67 ± 0.47	0.73 ± 0.53	-0.74	-0.23, 0.10	0.48
1.5	0.87 ± 0.58	1.05 ± 0.61	-1.81	-0.39, 0.02	0.08
2	1.04 ± 0.66	1.30 ± 0.67	-2.31	-0.48, -0.04	0.02
2.5	1.23 ± 0.65	1.51 ± 0.67	-2.59	-0.51, -0.07	0.01
3	1.38±0.67	1.66 ± 0.72	-2.35	-0.51, -0.04	0.02
3.5	1.51 ± 0.64	1.80 ± 0.75	-2.52	-0.53, -0.06	0.02
4	1.68 ± 0.63	1.95 ± 0.76	-2.25	-0.50, -0.03	0.02
4.5	1.81 ± 0.65	2.06 ± 0.79	-2.06	-0.50, -0.01	0.04
5	1.91 ± 0.70	2.15 ± 0.82	-1.88	-0.50, 0.01	0.06
5.5	2.08 ± 0.75	2.23 ± 0.82	-1.12	-0.41, 0.11	0.26
6	2.22 ± 0.77	2.33 ± 0.87	-0.77	-0.38, 0.17	0.43

**Figure 2:**
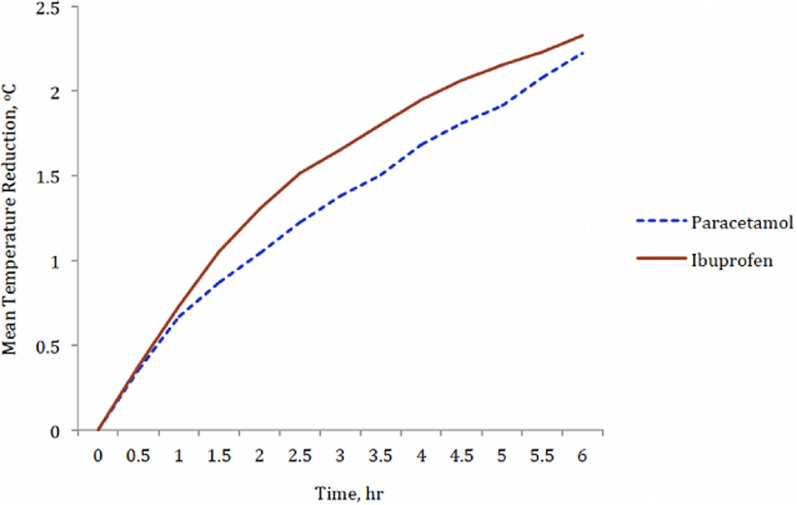
AUC for mean temperature reduction at different time points

**Mean temperature reduction assessed using area under the curve:** the mean temperature reduction was compared using the area under the curve (AUC) produced from baseline to the different time points up to 6 hours, for both ibuprofen and paracetamol groups. The time point (x-axis) was plotted against the mean reduction in temperature (y-axis) for both drugs (Figure 2). The comparison of the AUC for both drugs showed that the ibuprofen group had a greater AUC (9.08) than the paracetamol group (7.85).

**Proportion of afebrile children at different time points:** after the administration of the drugs at baseline, a greater proportion of children in the ibuprofen group had their temperature reduced to a normal level (temperature < 37.6°C) than in the paracetamol group at all observed time points (Table 3). This difference in the proportion of afebrile children between the groups was statistically significant at three time points: 1.5 hours (p = 0.04), 2 hours (p = 0.03) and 2.5 hours (p = 0.01). At the end of the first hour, two children in the ibuprofen group (2.9%) were observed to be afebrile while the children in the paracetamol group started becoming afebrile at 1.5 hours. The difference in the proportion of afebrile children between both groups was greatest at 2.5 hours (22%) and least at 0.5 hours (1.4%). At the end of six hours, eight children in the ibuprofen group were still afebrile while the paracetamol group had 11 afebrile children.

**Table 3:** comparison of proportion of afebrile children (temperature < 37.6°c) at different time points

Number and percentage of afebrile children					
Time, hr	Paracetamol group n (%)	Ibuprofen group n (%)	z-score	95% CI	p-value
0.5	0 (0)	1 (1.4)	-1.00	-0.04, 0.08	0.32
1	0 (0)	2 (2.9)	-1.42	-0.03, 0.10	0.16
1.5	5 (7)	13 (19)	-2.02	0.19, 0.23	0.04
2	13 (19)	24 (34)	-2.11	0.01, 0.30	0.03
2.5	17 (24)	32 (46)	-2.66	0.06, 0.35	0.01
3	31 (44)	41 (59)	-1.69	-0.02, 0.30	0.09
3.5	37 (53)	46 (66)	-1.55	-0.03, 0.28	0.12
4	44 (63)	51 (73)	-1.27	-0.05, 0.25	0.20
4.5	49 (70)	52 (74)	-0.57	-0.10, 0.19	0.57
5	51 (73)	59 (84)	-1.65	-0.02, 0.25	0.10
5.5	57 (81)	59 (84)	-0.45	-0.10, 0.15	0.65
6	59 (84)	62 (89)	-0.74	-0.07, 0.16	0.46

**Adverse events observed in the children:** the distribution of adverse events observed between both groups is shown in Table 4. This was not statistically significant between both groups (p = 0.19). Also, the paracetamol group had slightly greater odds (1.29) of developing adverse events than the ibuprofen group, which was not statistically significant (p = 0.12).

**Table 4:** distribution of adverse events observed

	Presence of adverse events, n (%)			Adverse events (Overall), n (%)			
Group	Vomiting	Diarrhoea	Excessive sweating	Present	Absent	χ2	OR (95%CI)
Paracetamol	5 (7.14)	3 (4.28)	2 (2.86)	10 (14.3)	60 (85.7)		
Ibuprofen	9 (12.9)	2 (2.96)	5 (7.14)	16 (22.9)	54 (77.1)	1.70	1.29 (-0.30, 1.45)

## Discussion

The study compared the antipyretic effect and safety of a single dose of ibuprofen 10mg/kg/dose and paracetamol 15mg/kg/dose given to febrile children aged six months to five years of age, followed by a six-hour observation period. Ibuprofen produced a greater antipyretic effect than paracetamol between 1.5 hours and 2.5 hours following administration of the medications. The mean temperature reduction measured at half-hourly intervals was higher for ibuprofen than paracetamol at all the time points. The area under the curve (AUC) was used to show the mean temperature reduction produced by the drugs from baseline to the end of the observation period. Ibuprofen produced a greater AUC than paracetamol with an effect size of 0.13 produced at six hours in this study. This magnitude of the difference in mean temperature reduction favoured ibuprofen as a more effective antipyretic agent. The difference in the fever reduction effects of ibuprofen and paracetamol observed in this study is consistent with a meta-analysis of 10 data sets for fever reduction in which Perrot *et al*. [10] concluded that ibuprofen was a more efficacious antipyretic agent than paracetamol in terms of maximum temperature reduction and the length of antipyretic action. They revealed that all point-estimates of the mean weighted-effect sizes for comparisons between ibuprofen and paracetamol were in favour of ibuprofen as a more efficacious antipyretic agent, with effect size values ranging from 0.19 to 0.33. The study by Vyas *et al*. [15] also found that ibuprofen was more efficacious in reducing fever than paracetamol in the four-hour observation period of the study. Our findings, however, differed from some previous studies which showed no difference in the antipyretic effects of ibuprofen versus paracetamol within six hours of drug administration. Seyfhashemi *et al*. [20] did not notice any difference in the mean temperature reduction between ibuprofen and paracetamol within one and six hours. He showed that ibuprofen had a greater antipyretic effect only after the sixth hour. paracetamol and ibuprofen were found to be similar in terms of overall antipyretic effect in a study by Seyfhashemi *et al*. [20]. Autret-Leca *et al*. [21] also found that both drugs had equivalent antipyretic effect in febrile children of ages between 3 months and 12 years.

A significantly higher proportion of children were afebrile between 1.5 hours and 2.5 hours post administration of the drugs in the ibuprofen group than the paracetamol group with a mean temperature of< 38.0°C achieved in the ibuprofen group by 2.0 hours of administration of the drugs as against 2.5 hours in the paracetamol group. This finding suggests that ibuprofen has a faster fever reducing effect and a more sustained action. Seyfhashemi *et al*. [20] in Iran among children of ages two months to 12 years showed that the proportion of afebrile patients at every time point was higher with ibuprofen than paracetamol throughout the 6-hour post-treatment period. The difference in the proportion of afebrile children between both groups was greatest at 2.5 hours in our study while Seyfhashemi *et al*. [20] recorded the greatest difference between the groups at the third hour. Vyas *et al*. [15] noticed the greatest difference in the proportion of afebrile children in the first hour in their study. The children in the ibuprofen group started becoming afebrile faster than the ones in the paracetamol group in our study. The faster fever reducing effect noticed with ibuprofen is similar to the finding by Hay *et al*. [22], which revealed that ibuprofen reduced fever faster than paracetamol in the first four hours. The adverse events observed in our study were mild and included vomiting, diarrhoea and excessive sweating. These events occurred more in the ibuprofen group than the paracetamol group but the difference between both groups was not statistically significant. Gastrointestinal symptoms like vomiting and diarrhoea are known adverse effects of NSAIDs such as ibuprofen [12]. The nature and severity of adverse events observed in this study were similar to those reported by Hay *et al*. [22]. The frequency of the adverse events in our study was more in the ibuprofen group but were equally distributed between both groups in the study by Hay *et al*. [22]. Similarly, Perrot *et al*. [10] observed mild adverse events such as sweating, nausea, vomiting and abdominal pain, which required no treatment. Lesko *et al*. [23] also did not observe any serious adverse event with short-term use of these antipyretics. A well powered study conducted over a reasonable length of time is required to fully appreciate the adverse event profile and severity of both drugs in underfives with fever.

## Conclusion

The mean temperature reduction of a single dose of ibuprofen at 10mg/kg was significantly better than that of paracetamol at a 15mg/kg between the 2.0 hours and 4.5 hours post administration of the medications. The proportion of afebrile children at the half-hourly time points was significantly higher in the ibuprofen than paracetamol group between 1.5 hours and 2.5 hours of administration of the drugs. Both drugs were associated with mild adverse events which were of similar profile.

### What is known about this topic

- Fever is a common symptom of most childhood illnesses in developing countries;
- The condition disrupts the child’s activity and it is of concern to most caregivers;
- Fever is usually treated with physical and pharmacological agents.

### What this study adds

- A single dose of ibuprofen was associated with a significant mean temperature reduction 2.0 hours-4.5 hours post administration when compared to paracetamol;
- A significantly higher proportion of children in the ibuprofen group were afebrile 1.5 hour-2.5 hours post administration of the medications when compared to the paracetamol group;
- Ibuprofen was associated with more adverse events than paracetamol though the difference was not statistically significant.
